# Posttraumatic growth, depression and posttraumatic stress in relation to quality of life in tsunami survivors: a longitudinal study

**DOI:** 10.1186/s12955-014-0202-4

**Published:** 2015-02-07

**Authors:** Johan Siqveland, Egil Nygaard, Ajmal Hussain, Richard G Tedeschi, Trond Heir

**Affiliations:** Department of Mental Health Services, Akershus University Hospital, R & D Mental Health Services, 1478 Lørenskog, Norway; Institute of Clinical Medicine, University of Oslo, Oslo, Norway; Norwegian Centre for Violence and Traumatic Stress Studies, Oslo, Norway; Department of Psychology, University of Oslo, Oslo, Norway; Department of Psychology, University of North Carolina at Charlotte, Charlotte, NC USA

**Keywords:** Natural disaster, Posttraumatic stress, Depression, Posttraumatic growth, Quality of life

## Abstract

**Background:**

Quality of life (QoL) may often be reduced in survivors of a natural disaster. This paper investigated how posttraumatic growth (PTG), depression and posttraumatic stress interact and independently predict QoL in a longitudinal study of disaster survivors.

**Methods:**

A total of 58 Norwegian adults who were present in Khao Lak, Thailand at the time of the 2004 Southeast Asia Tsunami completed self-report questionnaires 2 and 6 years after the disaster. The participants reported symptoms of depression and posttraumatic stress as well as PTG and QoL. Multiple mixed effects regression analyses were used to determine the independent effects of PTG, depression and posttraumatic stress on QoL measured 2 and 6 years after the disaster.

**Results:**

Posttraumatic stress and depression were negatively related to QoL. PTG was not significantly related to QoL in a bivariate analysis. However, considerable interaction effects were found. Six years after the tsunami, high levels of posttraumatic stress were related to lower QoL in those participants with low levels of PTG, whereas lower levels of depression were related to higher QoL in those participants with high levels of PTG.

**Conclusions:**

Posttraumatic stress and depression are negatively associated with QoL after a natural disaster. PTG may serve as a moderating factor in this relationship.

**Electronic supplementary material:**

The online version of this article (doi:10.1186/s12955-014-0202-4) contains supplementary material, which is available to authorized users.

## Background

Reduced quality of life (QoL) has been reported after catastrophic events, such as natural disasters [1-3], and may be related to material losses and somatic injuries as well as psychological distress. The relationship between psychological distress (e.g., posttraumatic stress and depression) and QoL has been consistently negative in the scientific literature. In reports from clinical trials, 59% of participants with posttraumatic stress disorder (PTSD) had QoL scores more than two standard deviations below the community norm [4]. However, all psychological changes related to disaster exposure may not be deleterious to QoL; one example of these psychological changes being posttraumatic growth (PTG). Intuitively, PTG (e.g., improved personal relationships, higher appreciation of life, increased spirituality, new possibilities and increased confidence in personal strength) may even be to be positively related to QoL. However, the relationship between PTG and QoL is more complicated. According to the most widely utilized model [5], PTG is related to posttraumatic stress and depression because some level of psychological distress is necessary for PTG to develop, an assertion which has been supported by previous research on disaster survivors [6]. Distress works as a catalyst in the process of cognitive restructuring that is needed to look at the world in new ways characteristic of PTG. Therefore, PTG and posttraumatic stress are not opposite ends of a continuum; instead, they exist on separate dimensions that are related through the level of distress experienced. Furthermore, it is suggested that this relationship may change over time: PTG results in a higher QoL when trauma survivors develop a sense of meaning, change their life priorities, and create new and valuable perspectives on living [7].

There have been many studies that have documented PTG reports of survivors of various natural disasters including hurricanes and earthquakes ([8-12]). Considering the relationship between PTG and QoL specifically, there are no studies of natural disasters. However, reports by survivors of breast cancer [13] and a meta-analysis [14], that pooled seven studies reported no main effect of PTG on QoL. Thus, we did not expect to find any main effect from PTG on QoL. Rather, we hypothesized that PTG would have a buffering effect on the relationships between posttraumatic stress and QoL and between depression and QoL. We expected that participants who reported high levels of posttraumatic stress or depression *and* high levels of PTG would report higher levels of QoL than those participants who reported high levels of posttraumatic stress or depression and simultaneously low levels of PTG.

## Methods

### Participants and procedure

**Figure 1:**
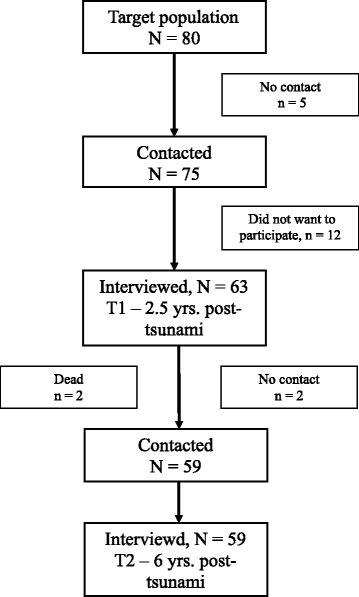
**Flowchart of the study population consisting of Norwegian tourists (≥18 years) in Khao Lak (Thailand) at the time of the disaster.**

After submitting an application to the Norwegian Data Inspectorate, the names of the Norwegian tourists who were present in the affected areas were made available for the present study (Figure 1). Out of the 80 Norwegian adults present in Khao Lak at the time of the tsunami, 75 were traceable and thus eligible for the present study. Out of these 75 persons, 63 agreed to participate in the study, were interviewed and completed self-report forms twice: 2 years (T1) and 6 years (T2) post-tsunami. The T2 data were collected by telephone interview. One participant did not reply to questions concerning QoL at T1, and 4 persons were lost to follow up at T2. Therefore, the present study’s sample consisted of 58 participants. For demographic background information and information regarding exposure, see Table 1. There were no significant differences between genders with respect to age, marital status, employment, education, disaster exposure or loss. A previous attrition analysis on the same sample showed that study participants and non-participants did not differ significantly in age or gender, and the most common reason given for not participating in the study was lack of time [15].

**Table 1:** **Demographic and exposure information and descriptive statistics for major study variables (** ***N*** **= 58)**

**Demographic and exposure variables**	**Prevalence**
Female gender	55.2%
Age (Mean/ SD)	40.5 (11.3)
Married/cohabiting	63,8%
Employed	69%
Higher education	69%
***Family members participating***	
1	34,5%
2	55.2%
3	10.3%
***Exposure***	
No direct exposure to waves	20.7%
Touched or chased by waves	39.7%
Caught by waves	39.7%
***Injury and loss***	
Were injured	39.7%
Hospitalized	19%
Lost family member	20.7%
**Major study variables**	**Mean (** ***SD*** **)**
Posttraumatic growth	50.6 (25.2)
Depression^a^	2.7 (4.2)
Posttraumatic stress^b^	31.6 (15.1)
General quality of life at T1	4.0 (0.9)
Health-related quality of life at T1	3.7 (1.2)
General quality of life at T2	4.1 (1.0)
Health-related quality of life at T2	3.7 (1.1)

Note: All variables were measured two years post-tsunami. In addition, quality of life was measured both two and six years post-tsunami.

^a^The level of depression as measured by the General Health Questionnaire.

^b^The level of posttraumatic stress as measured by the PCL.

At T1, none of the participants were involved in unsettled insurance cases related to the tsunami. There were no significant gender differences in our analysis of. We obtained written informed consent from all study participants before the interview began. The study was approved by the Regional Committee for Medical Research Ethics.

### Measures

PTG, depression and posttraumatic stress were measured at T1; QoL was measured at T1 and T2. Posttraumatic growth was assessed using the Posttraumatic Growth Inventory (PTGI) [16], which is composed of 21 items with six response alternatives (0 indicating no change, 5 indicating a high degree of change) and assesses changes on five PTG domains: relating to others, new possibilities, personal strength, appreciation of life, and spiritual change. The PTGI showed high internal reliability (Cronbach’s alpha .95) in the present sample.

The PTSD Checklist (PCL) [17] was used to measure posttraumatic stress at T1. The PCL is a 17-item self-administered questionnaire assessing the full domain of DSM-IV PTSD symptoms. We used the PCL-S (specific) version in which the symptoms endorsed were specifically linked to the tsunami. The participants rated each item on a 5-point Likert scale (1 = not at all, 2 = a little, 3 = moderately, 4 = quite a bit, 5 = extremely) based on the extent to which they have been bothered by the 17 symptoms within the last month; the total score of all 17 items (range 17 – 85) represents the person’s level of posttraumatic stress symptoms. The PCL-S has performed well in previous research studies on the same patient population [18] and has demonstrated high internal reliability in the present sample (Cronbach’s alpha .96).

The level of depression was measured at T1 using the General Health Questionnaire (GHQ-28) [19]. The GHQ-28 has four subscales: somatic symptoms, anxiety and insomnia, social dysfunction, and depression. Each item on the GHQ-28 has four response alternatives (scored from 0 to 3). The sum of the 7 items in the depression subscale was used to measure the level of depression; this measurement demonstrated high internal reliability (Cronbach’s alpha .93) in the present study.

QoL was measured at T1 and T2 using 2 items from the World Health Organization Quality of Life-Bref scale (WHOQOL-Bref) [20]. The scale has demonstrated good psychometric properties in Norwegian general population research [21]. Questions on the WHOQOL-Bref were answered with five-point rating scale responses, with 1 indicating the lowest QoL and 5 indicating the highest QoL. The first item measured global quality of life (G-QoL) using the question “How would you rate your quality of life?” The second item measured health-related quality of life (HR-QoL) using the question “How satisfied are you with your health?”

The T2 follow-up was conducted via telephone interviews. Due to time concerns QoL was therefore measured with single items. Previous unpublished analysis from the same material showed that on T1, where we measured QoL with the full WHOQOL-Bref scale, there was moderate to high correlations between the full scale score and the two questions selected for the present publication. The correlations ranged from .57 to .69 for G-QoL and from .42 to .59. for HR-QoL. Also T1 analysis with both the full scale score and the single items yielded the same conclusions.

### Statistics

Gender differences were analyzed with Pearson’s Chi-squared test for grouped variables and the Mann–Whitney U test for continuous variables. The bivariate relationships were calculated using Spearman’s correlation coefficients. Mixed effects regression analyses, with family as the random subject variable, were used to control for the confounding effects of shared family status in the bivariate and multiple analyses of predictors of G-QoL and HR-QoL.

Multiple mixed effects regression analyses were used to determine the independent effects of PTG, depression and posttraumatic stress at T1 on G-QoL and HR-QoL at T1 and T2. For comparability, the independent variables were standardized into Z-scores before being simultaneously entered into the statistical model. Theoretically, all main effects could influence each other. Therefore, the models were rerun with all of the main effects and possible two-way interaction effects between the main effects simultaneously entered into the models. The models predicting G-QoL and HR-QoL at T2 were rerun controlling for QoL at T1. The models were also repeated controlling for age, gender, exposure to waves and loss of family member.

There was no missing information among the 58 participants on any of the analyzed variables. A significance level of .05 was used for all statistical tests. All statistical analyses were conducted using IBM SPSS Statistics, version 19.0.

## Results

G-QoL and HR-QoL were moderately related at T1 (*r*_*s*_ = .62, *p* ≤ .001) and at T2 (*r*_*s*_ = .74, *p* ≤ .001) and were relatively stable from T1 to T2 (T1 and T2 correlations for G-QoL were .73 and .61 for HR-QoL, both *p* ≤ .001).

Neither gender nor age was significantly related to QoL (Table 2). Of the disaster-related variables, loss of family member and direct exposure to water were related to lower G-QoL at T1 but were not significantly related to G-QoL at T2 or to HR-QoL at T1 or T2.

**Table 2:** **Bivariate mixed effects analyses predicting QoL in Norwegian tourists** ***(N*** **= 58) at 2 (T1) and 6 (T2) years post-tsunami**

	**Global quality of life**						**Health-related quality of life**					
	**T1**			**T2**			**T1**			**T2**		
	**b**	**95% CI**	**p**	**b**	**95% CI**	**p**	**b**	**95% CI**	**p**	**b**	**95% CI**	**p**
**Gender**												
**Male**	−0.04	−0.40, 0.31	.80	−0.14	−0.53, 0.24	.45	−0.07	−0.51, 0.37	.74	0.04	−0.38, 0.46	.84
**Female** ^**a**^	0			0			0			0		
**Age at time of tsunami**	−0.01	−0.03, 0.01	.31	0.00	−0.02, 0.03	.69	−0.01	−0.04, 0.01	.28	−0.01	−0.04, 0.01	.28
**Loss of family member**												
**No**	0.79	0.16, 1.42	.02	0.68	−0.06, 1.42	.07	0.18	−0.72, 1.08	.69	0.52	−0.31, 1.35	.21
**Yes** ^**a**^	0			0			0			0		
**Caught by waves**												
**No**	1.02	0.28, 1.77	.009	0.75	−0.16, 1.66	.10	0.87	−0.16, 1.89	.09	0.90	−0.09, 1.88	.07
**Nearly or partly**	0.51	−0.09, 1.11	.09	0.30	−0.43, 1.04	.41	−0.38	−1.21, 0.45	.36	0.19	−0.61, 0.98	.63
**Completely** ^**a**^	0			0			0			0		
**Posttraumatic growth**	0.03	−0.16, 0.22	.73	0.04	−0.17, 0.26	.70	0.01	−0.23, 0.25	.94	0.15	−0.07, 0.37	.18
**Depression** ^**b**^	−0.52	−0.70, −0.34	< .001	−0.56	−0.76, −0.36	< .001	−0.59	−0.83, −0.35	< .001	−0.58	−0.82, −0.34	< .001
**Posttraumatic stress** ^**c**^	−0.55	−0.73, −0.36	< .001	−0.53	−0.75, −0.30	< .001	−0.47	−0.72, −0.22	< .001	−0.42	−0.69, −0.16	.002

Note: Multilevel regression analysis controlled for the effect of mutual family members. Figures are regression coefficients (95% confidence intervals in parenthesis). All predictors were measured two years post-tsunami. Posttraumatic growth, depression and posttraumatic stress were standardized before being entered into the model.

^a^Females, those without the loss of a family member and those who were completely caught by waves were set to have a mean of 0 in the mixed effects models.

^b^Level of depression as measured by the General Health Questionnaire.

^c^Level of posttraumatic stress as measured by the PCL.

PTG (Table 1) was not significantly related to demographic variables (gender, age), exposure or loss. PTG was weakly positively related to posttraumatic stress (*r*_*s*_ = .28, *p* = .03) but was not significantly related to depression. Depression and posttraumatic stress were significantly related *(r*_*s*_ = .55, *p* ≤ .001).

In the bivariate mixed effects analyses predicting QoL (Table 2), PTG was not statistically related to QoL at either time point, depression had a significant negative relationship with QOL at both T1 and T2, and posttraumatic stress at T1 was negatively related to QoL at both T1 and T2.

In the multilevel multiple regression analysis, in which depression, posttraumatic stress and PTG were simultaneously entered (Table 3), PTG was unrelated to G-QoL and HR-QoL at T1 and T2, depression was negatively related to T1 and T2 G-QoL and HR-QoL and posttraumatic stress negatively related to T1 and T2 G-QoL, but not significantly related to HR-QoL.

**Table 3:** **Multiple mixed effects analyses predicting QoL two and six years post-tsunami (** ***N*** **= 58)**

	**Global quality of life**						**Health-related quality of life**					
	**T1**			**T2**			**T1**			**T2**		
	**b**	**95% CI**	**p**	**b**	**95% CI**	**p**	**b**	**95% CI**	**p**	**b**	**95% CI**	**p**
***Models without interaction variables***												
**Posttraumatic growth**	0.13	−0.04, 0.29	.12	0.11	−0.09, 0.30	.27	0.08	−0.15, 0.31	.48	0.15	−0.09, 0.39	.20
**Depression** ^**a**^	−0.26	−0.47, −0.05	.02	−0.42	−0.68, −0.16	.002	−0.43	−0.73, −0.12	.007	−0.47	−0.78, −0.15	.004
**Posttraumatic stress** ^**b**^	−0.42	−0.64, −0.19	< .001	−0.27	−0.54, 0.00	.05	−0.27	−0.59, 0.05	.10	−0.18	−0.51, 0.14	.27
**Explained variance** ^**c**^	50.9%			44.3%			34.8%			37.9%		
**Model fit: AIC**	122.2			141.7			162.8			160.5		
***Models with interaction variables***												
**Posttraumatic growth**	0.13	−0.04, 0.30	.12	0.14	−0.05, 0.33	.14	0.13	−0.09, 0.35	.23	0.20	−0.03, 0.43	.09
**Depression** ^**a**^	−0.33	−0.60, −0.06	.02	−0.44	−0.75, −0.14	.005	−0.66	−1.01, −0.31	.001	−0.59	−0.95, −0.22	.002
**Posttraumatic stress** ^**b**^	−0.44	−0.68, −0.21	< .001	−0.39	−0.65, −0.14	.003	−0.42	−0.75, −0.09	.01	−0.26	−0.57, 0.06	.11
Depression* Posttraumatic stress	0.07	−0.10, 0.24	.40	0.08	−0.11, 0.26	.41	0.29	0.07, 0.52	.01	0.16	−0.07, 0.38	.17
Posttraumatic growth* Depression	−0.12	−0.35, 0.11	.30	−0.51	−0.77, −0.26	< .001	−0.17	−0.49, 0.15	.29	−0.39	−0.70, −0.08	.01
Posttraumatic growth* Posttraumatic stress	0.20	−0.04, 0.45	.10	0.49	0.22, 0.77	.001	0.22	−0.09, 0.54	.16	0.55	0.22, 0.89	.002
**Explained variance** ^**c**^	52.9%			58.3%			38.2%			47.4%		
**Model fit: AIC**	128.2			136.8			162.7			156.4		

Note. Multilevel regression analysis controlled for the effect of mutual family members. All variables were simultaneously entered into the regression model. Posttraumatic growth, depression and posttraumatic stress were standardized before being entered into the model. Figures are regression coefficients (95% confidence intervals in parenthesis). All predictors were measured two years post-tsunami. Explained variance is the percentage reduction in unexplained variance compared to a model without any independent variables. AIC for an empty model was 150.2 and 162.9 for general quality of life at T1 and T2, respectively, and 178.0 and 171.2 for health-related quality of life at T1 and T2, respectively.

AIC = Akaike’s information criterion.

*Interaction between variables.

^a^Level of depression as measured by the General Health Questionnaire.

^b^Level of posttraumatic stress as measured by the PCL.

^c^Variance within families was not possible to estimate for quality of life at T2. However, a multiple linear regression analysis without controlling for common family members gave identical estimates and p-values for fixed effects as the mixed effects model.

**Figure 2:**
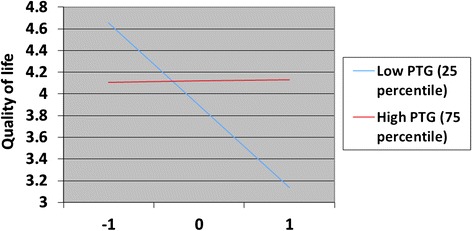
**Moderator effects of posttraumatic growth on the relationship between posttraumatic stress reactions and G-QoL.** The figure displays the moderator effects of posttraumatic growth on the relationship between posttraumatic stress reactions and general QoL at T2. Posttraumatic stress is standardized (Z-value).

**Figure 3:**
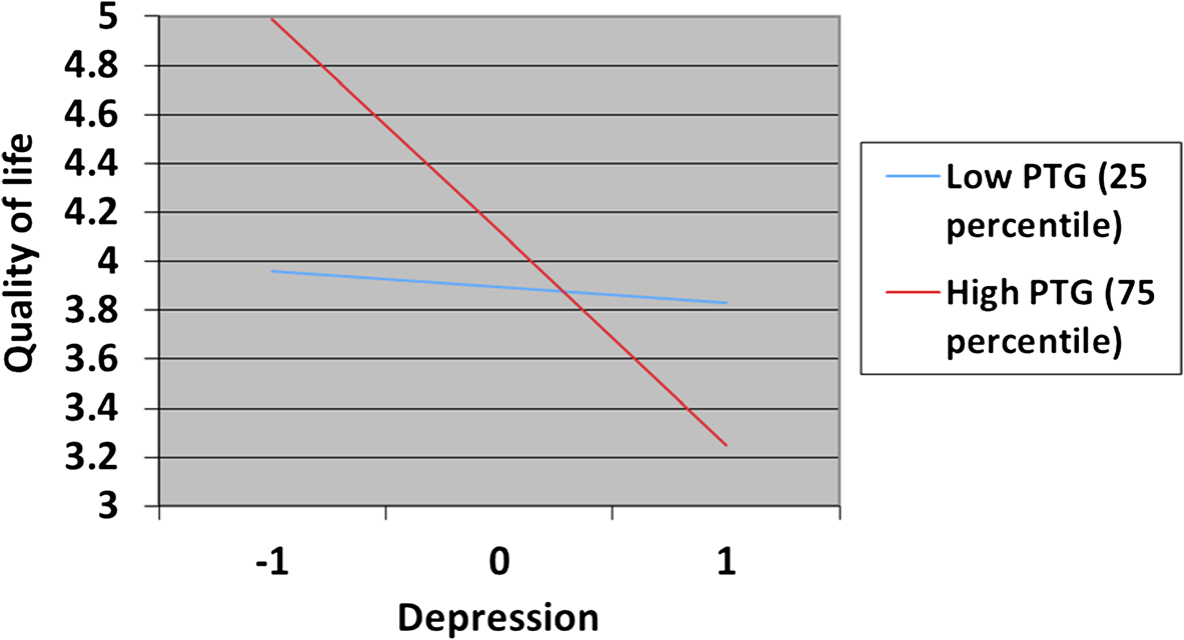
**Moderator effects of posttraumatic growth on the relationship between depression and G-QoL.** The figure displays the moderator effects of posttraumatic growth on the relationship between depression and general QoL at T2. Depression is standardized (Z-value).

Posttraumatic growth, depression and posttraumatic stress may have moderated each other’s effects on QoL. Therefore, analyses were repeated with the three possible two-way interaction effects included in the model. Explained variance at T1 increased only marginally when including these interaction effects (∆R2 = 2.0% and 3.4% for G-QoL and HR-QoL, respectively). However, explained variance at T2 increased substantially when including these three interaction effects in the models (∆R2 = 14.0% and 9.6% for G-QoL and HR-QoL, respectively). The interaction effect between depression and posttraumatic stress was significant only in the model predicting HR-QoL at T1. The interaction effects between PTG and depression, as well as between PTG and posttraumatic stress, were not significant at T1. However, both of these interaction effects were highly related to G-QoL and HR-QoL at T2. The interaction effect between PTG and posttraumatic stress was positively related to both G-QoL and HR-QoL at T2. Figure 2 shows how PTG may moderate the relationship between posttraumatic stress and QoL. Figure 3 shows how PTG may moderate the relationship between posttraumatic stress and QoL.

However, an opposite effect was found with respect to the interaction between PTG and depression. This interaction was negatively related to both G-QoL and HR-QoL at T2. Figure 2 shows how PTG may moderate the relationship between depression and QoL. Depression was negatively associated with QoL only for those who had high levels of PTG.

When depression was defined at the 75th percentile, the main effect of PTG was not significant. Thus, there was a significant positive effect of PTG on QoL for those with low levels of depression (left side of Figure 3) but not for those with high levels of depression (right side of Figure 3).

When G-QoL at T1 was entered into the model predicting G-QoL at T2 (including interaction effects), the only main effect was G-QoL at T1 (*B* = 0.57, 95% *CI* = 0.31 to 0.83, *p* < .001). Similarly, in the other analysis with HR-QoL at T2 as the outcome, the only main effect seen was the T1 measurement of HR-QoL (*B* = 0.35, 95% *CI* = 0.11 to 0.58, *p* = .005). However, the interaction effects between PTG and depression, as well as between PTG and posttraumatic stress, were still associated with QoL in the same direction presented in Figures 2 and 3.

Similar models as presented in Table 3 (both with and without interaction effects) were also reanalyzed controlling for covariates such as gender, age and exposure (loss of family member and caught by the waves), (Additional file 1: Table S1), with similar results as the conclusions presented above.

## Discussion

The relationship between posttraumatic stress, depression and posttraumatic growth and quality of life were investigated longitudinally in a group of adult Norwegian tourists who experienced the 2004 Southeast Asia Tsunami. To our knowledge, this is the first investigation of this relationship in disaster survivors who were evacuated to their homes shortly after the disaster, thus mostly escaping secondary stressors such as material loss. However, previous research have identified increased rates of PTSD in tourists experiencing the tsunami, and the event clearly had a psychological impact on this population [22]. As expected, posttraumatic stress and depression showed significant negative relations to QoL; in contrast, PTG was not significantly associated with QoL. Hence, persons who reported that the disaster brought about positive personal changes did not report higher levels of QoL. These findings are in line with most previous research studies on the relationship between QoL and PTG [14] as well as with the previously established negative relationships between QoL and measures of psychopathology [1,2].

One specific objective of this study was to investigate the interaction effects between PTG and posttraumatic stress and depression on QoL. Based on previous research studies [13], we expected that PTG would have a buffering effect on the relationship between posttraumatic stress and QoL as well as between depression and QoL. The results of our study partly met these expectations. We found the expected buffering effect of PTG on the relationship between posttraumatic stress and QoL. This finding could be interpreted as PTG serving as a coping resource or being the expression of a more balanced world view after the disaster, in which both positive and negative experiences are integrated.

However, we found no buffering effect of PTG on the relationship between depression and QoL. This finding could be interpreted in reference to the Janus Face Model of PTG [23]. Persons with high levels of depression may attempt to cope with this depression with a self-soothing type of PTG that is not related to positive psychological adjustment. People with posttraumatic stress symptoms, on the other hand, may experience more of what Zoellner and Maercker [23] refer to as veridical PTG, which is related to psychological adjustment and therefore QoL.

Another interesting finding is that the interaction between PTG and depression, as well as the interaction between PTG and posttraumatic stress, is not significant at T1; however, they are highly significant at T2. This may indicate that the process of PTG takes some time to effect psychological change in other areas. The model of PTG [5] posits that there are several series of social, cognitive, and emotional changes that may be necessary before substantial PTG is reported. Although it may seem that two years is a sufficient time frame for these processes, the results of this study may indicate otherwise. However, because PTG was not measured at T2, we cannot say if PTG changed over the course of this study. The development of PTG after the tsunami may have allowed for a better QoL in later years through a sense of resolution and meaning, or perhaps PTG allowed for particular positive changes in life (e.g., interpersonal relationships).

The present study has some limitations. The sample size was relatively small, increasing the risk of the model capitalizing on random fluctuations. We measured QoL with single-item questions which had a moderate to strong relationship to the full global QoL and health related QoL sub-scales. Measurements with more items measuring global and health related QoL and social relationships and psychological aspects of QoL, may have led to more robust conclusions. PTG was measured four years before the measurement of QoL and we did not control for traumatic or other types of events taking place in these intervening years. This may have introduced biases making our finding more difficult to interpret. Our sample population was exposed to a single discrete event, and then, participants were quickly removed and sent to their unaffected home communities in Norway. Thus, our sample did not experience secondary disaster stressors, such as destroyed communities and loss of property and livelihood. Therefore, caution should be used when generalizing the results of this study to other types of natural disasters [24].

In summary, this study replicates the well-established negative relationship between psychological distress and QoL. It also presents a more nuanced picture of the relationship between PTG and QoL. PTG, while not having any direct relationship with QoL, may be an important moderating factor on the relationship between psychological distress and QoL after natural disasters.

## Additional file

Additional file 1: Table S1. Multiple mixed effects analyses predicting QoL two and six years post-tsunami when controlling for gender, age and exposure (*N* = 58).
