# Supplementary material for: Posttraumatic growth, depression and posttraumatic stress in relation to quality of life in tsunami survivors: a longitudinal study
Source: Health Qual Life Outcomes. 2015 Feb 7;13:18. doi: 10.1186/s12955-014-0202-4 (PMC4326430; doi:10.1186/s12955-014-0202-4)
Supplement: Additional file 1: Table S1. — Multiple mixed effects analyses predicting QoL two and six years post-tsunami when controlling for gender, age and exposure (N = 58). [file 12955_2014_202_MOESM1_ESM.doc]

Additional file 1: Table S1 Multiple mixed effects analyses predicting QoL two and six years post-tsunami when controlling for gender, age and exposure (*N* = 58)

|  | **Global quality of life** | | | | | | **Health-related quality of life** | | | | | |
| --- | --- | --- | --- | --- | --- | --- | --- | --- | --- | --- | --- | --- |
|  | T1 |  |  | T2 |  |  | T1 |  |  | T2 |  |  |
|  | b | 95% CI | p | b | 95% CI | p | b | 95% CI | p | b | 95% CI | p |
| ***Models without interaction variables*** |  |  |  |  |  |  |  |  |  |  |  |  |
| **Posttraumatic growth** | 0.14 | -0.02, 0.31 | .09 | 0.10 | -0.10, 0.30 | .33 | 0.11 | -0.12, 0.35 | .32 | 0.21 | -0.03, 0.45 | .09 |
| **Depression^a^** | -0.20 | -0.43, 0.02 | .08 | -0.39 | -0.67, -0.11 | .007 | -0.40 | -0.72, -0.08 | .01 | -0.42 | -0.74, -0.09 | .01 |
| **Posttraumatic stress^b^** | -0.40 | -0.64, -0.16 | .001 | -0.26 | -0.55, 0.03 | .08 | -0.30 | -0.64, 0.03 | .08 | -0.11 | -0.45, 0.24 | .54 |
| **Explained variance^c^** | 50.5% |  |  | 37.6% |  |  | 41.5% |  |  | 33.6% |  |  |
| **Model fit: AIC** | 129.7 |  |  | 150.4% |  |  | 163.1 |  |  | 165.3 |  |  |
| ***Models with interaction variables*** |  |  |  |  |  |  |  |  |  |  |  |  |
| **Posttraumatic growth** | 0.14 | -0.03, 0.32 | .10 | 0.11 | -0.09, 0.32 | .26 | 0.15 | -0.07, 0.38 | .18 | 0.23 | -0.01, 0.47 | .06 |
| **Depression^a^** | -0.23 | -0.52, 0.05 | .11 | -0.37 | -0.70, -0.05 | .03 | -0.62 | -1.00, -0.24 | .002 | -0.53 | -0.92, -0.14 | .009 |
| **Posttraumatic stress^b^** | -0.43 | -0.68, -0.18 | .001 | -0.41 | -0.68, -0.13 | .005 | -0.43 | -0.77, -0.08 | .02 | -0.23 | -0.57, 0.12 | .19 |
| **Depression*Posttraumatic stress** | 0.05 | -0.13, 0.22 | .60 | 0.05 | -0.14, 0.24 | .60 | 0.26 | 0.03, 0.49 | .03 | 0.16 | -0.07, 0.40 | .17 |
| **Posttraumatic growth*Depression** | -0.14 | -0.38, 0.10 | .26 | -0.53 | -0.80, -0.26 | < .001 | -0.17 | -0.50, 0.15 | .29 | -0.39 | -0.72, -0.07 | .02 |
| **Posttraumatic growth*Posttraumatic stress** | 0.19 | -0.07, 0.45 | .14 | 0.54 | 0.25, 0.84 | .001 | 0.20 | -0.14, 0.54 | .25 | 0.50 | 0.15, 0.85 | .007 |
| **Explained variance^c^** | 50.8% |  |  | 56.7% |  |  | 43.5% |  |  | 42.6% |  |  |
| **Model fit: AIC** | 136.0 |  |  | 145.3 |  |  | 164.5 |  |  | 163.4 |  |  |

Note. Multilevel regression analysis controlled for the effect of mutual family members. All models included the following control variables; gender, age at time of the tsunami, loss of family member and whether they were caught by the waves (no, nearly/partly, completely). None of these control variables were significant in any of the multiple models, and are not shown in the table for readabilities sake. All variables were simultaneously entered into the regression model. Posttraumatic growth, depression and posttraumatic stress were standardized before being entered into the model. Figures are regression coefficients (95% confidence intervals in parenthesis). All predictors were measured two years post-tsunami. Explained variance is the percentage reduction in unexplained variance compared to a model without any independent variables. AIC for an empty model was 150.2 and 162.9 for general quality of life at T1 and T2, respectively, and 178.0 and 171.2 for health-related quality of life at T1 and T2, respectively.

AIC = Akaike’s information criterion.

^a^ Level of depression as measured by the General Health Questionnaire.

^b^ Level of posttraumatic stress as measured by the PCL.

^c^ Variance within families was not possible to estimate for quality of life at T2. However, a multiple linear regression analysis without controlling for common family members gave identical estimates and p-values for fixed effects as the mixed effects model.
